# MegaR: an interactive R package for rapid sample classification and phenotype prediction using metagenome profiles and machine learning

**DOI:** 10.1186/s12859-020-03933-4

**Published:** 2021-01-18

**Authors:** Eliza Dhungel, Yassin Mreyoud, Ho-Jin Gwak, Ahmad Rajeh, Mina Rho, Tae-Hyuk Ahn

**Affiliations:** 1 Program in Bioinformatics and Computational Biology, Saint Louis University, Saint Louis, MO USA; 2 Department of Computer Science and Engineering, Hanyang University, Seoul, Korea; 3 Department of Computer Science, Saint Louis University, Saint Louis, MO USA

**Keywords:** Metagenomics, Machine learning, R-package, Phenotype prediction, Sample classification

## Abstract

**Background:**

Diverse microbiome communities drive biogeochemical processes and evolution of animals in their ecosystems. Many microbiome projects have demonstrated the power of using metagenomics to understand the structures and factors influencing the function of the microbiomes in their environments. In order to characterize the effects from microbiome composition for human health, diseases, and even ecosystems, one must first understand the relationship of microbes and their environment in different samples. Running machine learning model with metagenomic sequencing data is encouraged for this purpose, but it is not an easy task to make an appropriate machine learning model for all diverse metagenomic datasets.

**Results:**

We introduce MegaR, an R Shiny package and web application, to build an unbiased machine learning model effortlessly with interactive visual analysis. The MegaR employs taxonomic profiles from either whole metagenome sequencing or 16S rRNA sequencing data to develop machine learning models and classify the samples into two or more categories. It provides various options for model fine tuning throughout the analysis pipeline such as data processing, multiple machine learning techniques, model validation, and unknown sample prediction that can be used to achieve the highest prediction accuracy possible for any given dataset while still maintaining a user-friendly experience.

**Conclusions:**

Metagenomic sample classification and phenotype prediction is important particularly when it applies to a diagnostic method for identifying and predicting microbe-related human diseases. MegaR provides various interactive visualizations for user to build an accurate machine-learning model without difficulty. Unknown sample prediction with a properly trained model using MegaR will enhance researchers to identify the sample property in a fast turnaround time.

## Background

Metagenomics, studying microbial community and diversity from environmental samples directly without culture, is applied in many research projects for last two decades aiming to understand microbes’ impact on human, animal, plant, ocean, and environmental niches [1].

The human microbiota is the aggregated clusters of microorganisms that colonize on exposed surfaces such as skin, respiratory tract, and gastrointestinal tract. Human-microbes projects such as MetaHIT consortium and human microbiome project (HMP) sought to study microorganism diversity in and on healthy or sick cohorts via advanced metagenomic sequencing techniques [2, 3]. Not only human but also diverse ecosystems with microbes were studied using metagenomics including Tara Oceans project that is another big consortium to investigate the ocean microbiome to understanding its functional role at a global scale [4]. These large-scale metagenomics projects provide huge publicly available datasets. Deep analysis of such metagenomic datasets can reveal the secret of interactions between microbes and hosts in nature.

Analyzing metagenomic data is challenging because a sample can contain thousands of species, each with differing abundances, and multiple copies of the genomic sequences are sheared and fragmented as reads. The most often used technique to analyze the composition and diversity of microbes is 16S rRNA gene amplicon analysis that amplifies the 16S rRNA region to distinguish substantially identified gene regions [5]. Taxonomic assignment relies on the association of a specific 16S rRNA gene with a taxon; these associations are defined as operational taxonomic units (OTUs). Since OTUs are most commonly analyzed at the phyla or genera resolution, 16S rRNA sequencing technologies have a limited scope in analyzing microbial communities at the species and strain level. More recently, whole genome shotgun sequencing (WGS) has been adopted to increase sequence read depth and extend the range of capture to species level resolution and other microbes including viruses [6]. With extensive coverage provided, WGS allows for a more diverse picture of the microbes at the species and even strain level. Both sequencing techniques are currently being used to study the microbial landscape and have been evaluated for their inherent strengths and weaknesses [7]. The choice of 16S rRNA sequencing or WGS usually depends on the nature of the study: 16S is proper for large-scale analysis of a many samples such as longitudinal research and WGS provides a greater potential for higher resolution by identifying strains and even viruses that the 16S approach cannot. To address this technology gap, new advanced sequencing techniques are continuing to be developed and evaluated including shallow shotgun sequencing [8].

Taxonomy classification in metagenomics refers to identifying microbial genomes from closely related organisms in the metagenomic samples. QIIME (QIIME 2) is a widely used tools to analyze 16S rRNA gene sequences using OTU binning method from microbial communities [9]. In WGS, taxonomic profiles are examined by searching reads against reference genomes [10–12], analyzing *k*-mer frequency of reads [13, 14], or aligning reads with clade-specific marker genes including MetaPhlAn2 [15, 16]. A variety of tools including de novo assemblers, strain-level profilers, and functional analysis tools are also intensively used in metagenomics research [17–21]. Recently studied metagenomic research and related software tools are advanced compared to the standard metagenomics protocol such as identifying and quantifying microbial community composition. For example, the DIABIUMME project was designed to study the interactions and development of microbes, immune system, and diseases [22–24]. Another interesting metagenomic project is MetaSub designed for studying urban microbiomes their differences in the largest metro system in the world [25]. These types of research and challenges can be solved by investigating microbial patterns of the samples. Machine and deep learning techniques hold great promise in identifying such microbial patterns of the samples effectively and precisely [26, 27].

There were several machine-learning-based software tools to analyze the relationship of microbial sequencing data and sample phenotype. MetAML utilizes microbiome features by means of different machine learning classifiers to study the association of the microbes and phenotypes [28]. MetaDprof fits smoothing spline regression model to identify differential abundances of samples [29]. MetaLonDa is an R package that is able to identify substantial time intervals of way different abundant microbial features in longitudinal studies [30]. MetaNN provides a neural network classifier to identify host phenotypes from metagenomic data [31].

Such proposed software tools provide some advantages for microbiome-phenotype association prediction, but there are some limits. MetAML only supports WGS data, not 16S rRNA data set analysis. 16S rRNA sequencing is still mostly used, and is a powerful sequencing technique in metagenomics, it is important to provide a tool utilizing both 16S and WGS data. MetaDprof and MetaLonDa can effectively perform for the data sets from longitudinal studies, but they were not designed for general classifications of samples for phenotype prediction. MetaNN only utilized only 16S rRNA sequences, not WGS sequences [31].

We therefore developed MegaR (https://github.com/BioHPC/MegaR) to study microbiome-phenotype associations effectively and precisely including disease prediction capability. Our proposed framework MegaR has the following three main contributions:

1. MegaR supports both 16S rRNA and shotgun metagenomic sequencing data and can generate a model using different taxon level and different machine learning techniques.
2. MegaR provides user-friendly features for data preprocessing, model development, and model cross validation by power of interactive web-supporting library, R-Shiny,
3. MegaR classifies and predicts unknown samples based on the developed model precisely and speedily.

In this study, three different studies of DIABUMME project were used to assess the independent prediction accuracy of models for both 16S and WGS data and to compare strategies for practical use of the microbiome as a prediction tool. We also provide benchmark results of MegaR against MetAML using the data sets provided by MetAML to show the model accuracy, effectiveness, and user-friendly fine-tuning options to generate an optimized model with just a few clicks.

## Implementation

### MegaR data input

**Fig. 1:**
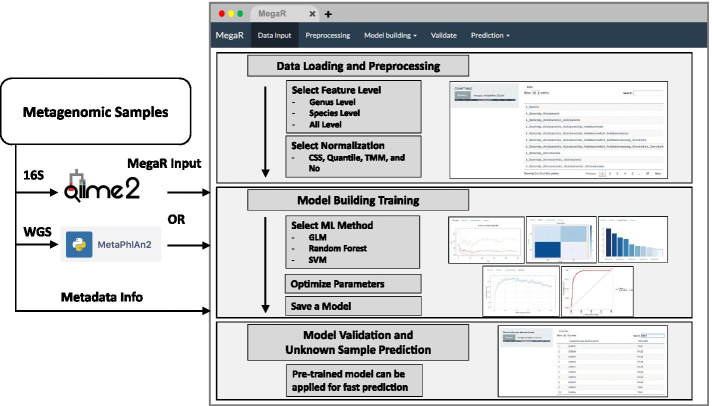
Illustration of MegaR flowchart. MegaR takes any taxonomy profiles from 16S rRNA and shotgun metagenomic data. After selecting taxonomic features, machine learning method, and multiple options, the user can train a model. Cross validation is supported before predicting unknown samples

We developed MegaR as an R package that uses 16S or whole genome taxonomic profile data sets to train a machine learning model for classification of unknown profiles. An overview of the MegaR pipeline can be seen in Fig. 1. For testing our package, we used two widely used taxonomy profiling tools QIIME (QIIME 2) [9] for 16S rRNA data and MetaPhlAn2 [15] for whole metagenome data. QIIME suite is one of the major software tools for 16S rRNA microbiome analysis. QIIME takes raw sequencing data from users, preprocesses the data, identify OTUs, and assigns taxons. MetaPhlAn2 takes meta-genomic shotgun sequencing data to profile the composition of microbial communities by mapping the sequences to the built-in clade specific maker genes. Both QIIME and MetaPhlAn2 have been used in many microbiome research projects including well investigated HMP and DIABIUMME projects. Most metagenome taxonomic profilers including QIIME and MetaPhlAn2 generate taxonomy profile output as OTU table or BIOM (Biological Observation Matrix) format [32] by providing simple scripts to merge multiple taxonomy profiles together. MegaR takes a merged OTU table or BIOM format as input. The user will also need to provide a metadata file containing the class of each sample in the data set.

### Machine learning methods

For the machine learning models in MegaR, we incorporated three machine learning classifiers: the first is generalized linear model (GLM), the second is support vector machine (SVM), and the third is random forest (RF). These approaches were implemented into MegaR by integrating the caret [33] and randomForest [34] packages.

The general linear model (GLM) is a statistical linear model also called as multivariate regression model [35]. GLM has several advantages over the other machine learning models. One is that it is easier to interpret since the coefficients are used within the mode. Many other accurate prediction models could be used to reduce error rates, but the ability of GLM to provide clarity while maintaining efficacy is the reason that this model has been adopted into MegaR.

A support vector machine (SVM) is a widely used non-probabilistic supervised machine learning method that tries to find an optimal hyperplane with maximizing the margin around the separation by the hyperplane [36]. SVM supports both linear and non-linear classification based on labeled data. SVM structure shapes a hyperplane or hyperplanes in multi-dimensional space to separate the data points with maximum margin classifier.

Random forest (RF) is also a commonly used decision-tree based method for classification and regression due to high-accuracy in prediction [34]. Random forest establishes numerous decision trees that are trained by the bagging method and select the features randomly. The primary benefit of using the RF method is providing pretty strong prediction accuracy in general by not overfitting with many trees. Another important advantage of using the RF model is that important features can be pulled and extracted easily. Those important features can be crucial role in many research studies such as identifying target associated features and biomarkers.

### Data processing and model development

The quantitative microbiome profiles in genus, species, and all levels can be selected by the user as features in the machine learning model. Because metagenomic datasets usually have different sizes and depths of sequences, MegaR provides four normalization options including Cumulative Sum Scaling (CSS), Quantile, Trimmed Mean of M-values (TMM), and none (NO) to normalize the aggregated metagenomic counts among samples [37]. The package also allows the user to set a minimum abundance threshold to filter out low abundance microbes that may not provide useful information.

**Fig. 2:**
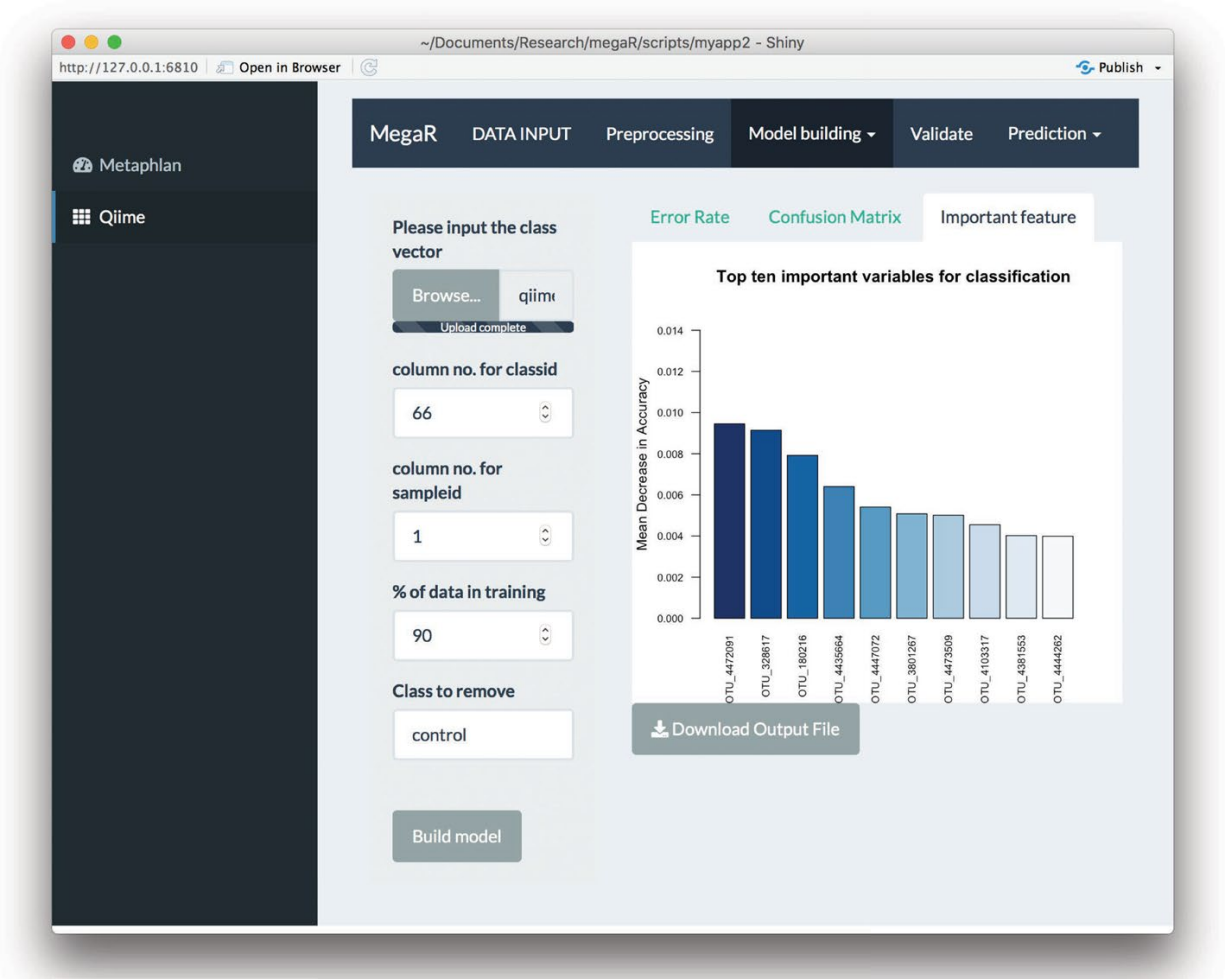
MegaR tool snapshot. Model building snapshot with *Important Feature* tab

After selecting the appropriate machine learning method for classification and modifying the parameters to best fit the data, the user can generate a model. MegaR provides an error rate for each prediction model generated that can be found under the *Error Rate* tab. The error rate of prediction on a test set is a better estimate of model accuracy, which can be estimated using a confusion matrix that is generated by the program under the *Confusion Matrix* tab. MegaR also provides an AUC graph of the model under the *AUC* tab. From a practical perspective, it is important to identify features that are useful in identifying the class of metagenomic samples. MegaR provides this data as a list of the top ten most important species or genera that are crucial in identifying the class of sample along with their variable importance under the *Important Feature* tab (Fig. 2). An additional feature of MegaR is “Class to remove” option that can improve prediction accuracy. When more than two classes are present in a data set, it is possible for the user not consider a specific class of the data set. Disregarding a class can also increase the prediction accuracy by narrowing down of the features.

### Cross validation

Cross-validation is a manner to access, judge, and review the performance of machine learning models. First and foremost, cross validation is essential to validate the model accuracy and model bias. This implies that the developed model should not be overfitted and not having bias.

To make a better model, all data set is not usually used for the training purpose, but split into training and validating/testing sets. For example, in *k*-fold cross validation, the dataset is shuffled and divided into *k* sub samples. The *k* − 1 samples are used as a training dataset and the single partition is used for validation. This process is repeated *k* times to represent the model performance. MegaR provides cross validation options allowing for an accurate prediction measure. The variance in fitting the model tends to be higher if it is fitted to a small dataset, therefore k-fold cross validation can have a high variance. MegaR provides users to select *N* independent runs of the tenfold cross validation to minimize such a high variance.

### Sample prediction

MegaR provides a *Prediction* tab for users to upload unknown samples and get a prediction on which category the unknown samples fall into among classes. Once a satisfactory model is created for the data set, the user can load a set of unknown samples into MegaR. Then MegaR generates a classification prediction for each sample in the set of classes, categories, or states. This function is useful for identifying the disease states of in individual which can provide a path towards precision medicine through the use of microbe composition as diagnostic biomarkers. MegaR also has a feature that allows the user to download the trained model for later use in *Prediction*. If a user clicks the Download Model button after training, the model (RDS type) file is generated and downloaded. The user can then load this model for prediction of unknown samples without re-training the model.

## Results

### Dataset

In order to demonstrate the efficacy of MegaR as a disease sample prediction tool, DIABIMMUNE (https://pubs.broadinstitute.org/diabimmune) microbiome project data sets were used to perform a sample pipeline execution in MegaR. The DIABIMMUNE project aims to find if the limitation in early exposure to bacteria and infections in western and developing country is related to increasing incidence of both autoimmune and allergic diseases. The DIABIMMUNE project provides three publicly available data sets.

The first dataset consists of 812 metagenomics samples and 1584 16S samples from three different countries: Estonia, Finland and Russia. Some samples that did not have labels were dropped. We also analyzed 16S rRNA datasets, with 448 from Finland and 664 from Russia. The second cohort, named T1D cohort, consists of 126 metagenomic samples from 19 children in Estonia and Finland: 92 samples with T1D; 32 samples without T1D; 2 samples without T1D status were filtered out. This cohort consists of 28 samples from birth to age one, 62 samples from age one to two and 38 samples from age two to three. In the T1D cohort there were 777 16S rRNA samples, out of which 175 had T1D, and 85 samples did not have T1D. 314 samples were from children from birth to one age, 297 samples were from children from one to two, and 166 children were from two to three in terms of the age group. The third cohort is called antibiotic cohort and consists of 240 metagenomic samples, from 39 subjects. There were 139 samples from children who were treated antibiotics and 101 samples from children who were not treated with antibiotics. In the 16S rRNA data set, there are 528 samples from children who were not treated with antibiotics while 520 samples were from children who were treated with antibiotics.

In order to benchmark the performance of MegaR against other packages, we used the data set from the MetAML project [28]. From this dataset, we compared the performance of MegaR against MetAML for the T2D [38, 39] and Cirrhosis [40] data sets. The T2D dataset used was an aggregated dataset from two separate studies, totaling 490 participants, 345 being Chinese and 145 being European. Samples were obtained from fecal samples in these studies. The liver cirrhosis data set consists of 98 patients and 83 control individuals.

### Taxonomy profiling

Preprocessed shotgun metagenomic data sets were downloaded from the DIABIMMUNE project. Left and right paired-end reads were concatenated together. The resulting data was run through MetaPhlAn2 using the parameter -t rel_ab_w_read_stats to obtain relative abundance and the number of reads derived from each clade. This estimate was extracted from each sample and merged into one file. The MetaPhlAn2 table that is available at the DIABIMMUNE project website had relative abundance as feature value. Our test shows that estimate of count as generated by MetaPhlAn2 option above is much better for classification. The associated metadata file was downloaded from the DIABIMMUNE website. All 16S rRNA taxonomy profiles were downloaded as an OTU table in BIOM format or tab separated format from the DIABIMMUNE project website.

### Model and prediction accuracy

We used MegaR to analyze different datasets from the DIABIMMUNE research group. In the preliminary research, we tested each machine learning model available in MegaR; GLM, SVM and RF for each dataset (Table 1). Overall the model was tenfold more accurate when species was selected for feature rather than genus. So, all our analysis thereof uses species for the analysis. In the case of RF, the model is more accurate for WGS data than 16S rRNA data in the three-country cohort and the T1D cohort. In the case of SVM and GLM, all models from 16S rRNA metagenomics had a higher accuracy than WGS. Among RF, SVM and GLM, RF performed the best followed by SVM and GLM, with the exception of the 16S rRNA T1D cohort where SVM performs the best followed by RF and GLM.

**Table 1:** Accuracy of RF, SVM and GLM across three datasets from DIABIMMUNE research group

Dataset	Data type	Accuracy		
		RF	SVM	GLM
Three country cohort	16S	**0.8832**	0.8063	0.7844
	WGS	**0.8846**	0.7014	0.5165
T1D cohort	16S	0.9017	**0.9245**	0.5897
	WGS	**0.9385**	0.7719	0.6316
Antibiotic cohort	16S	**0.8666**	0.7544	0.7397
	WGS	**0.7149**	0.6328	0.5362

Bold values represent the highest accuracy for each type of tested data in any given dataset

**Fig. 3:**
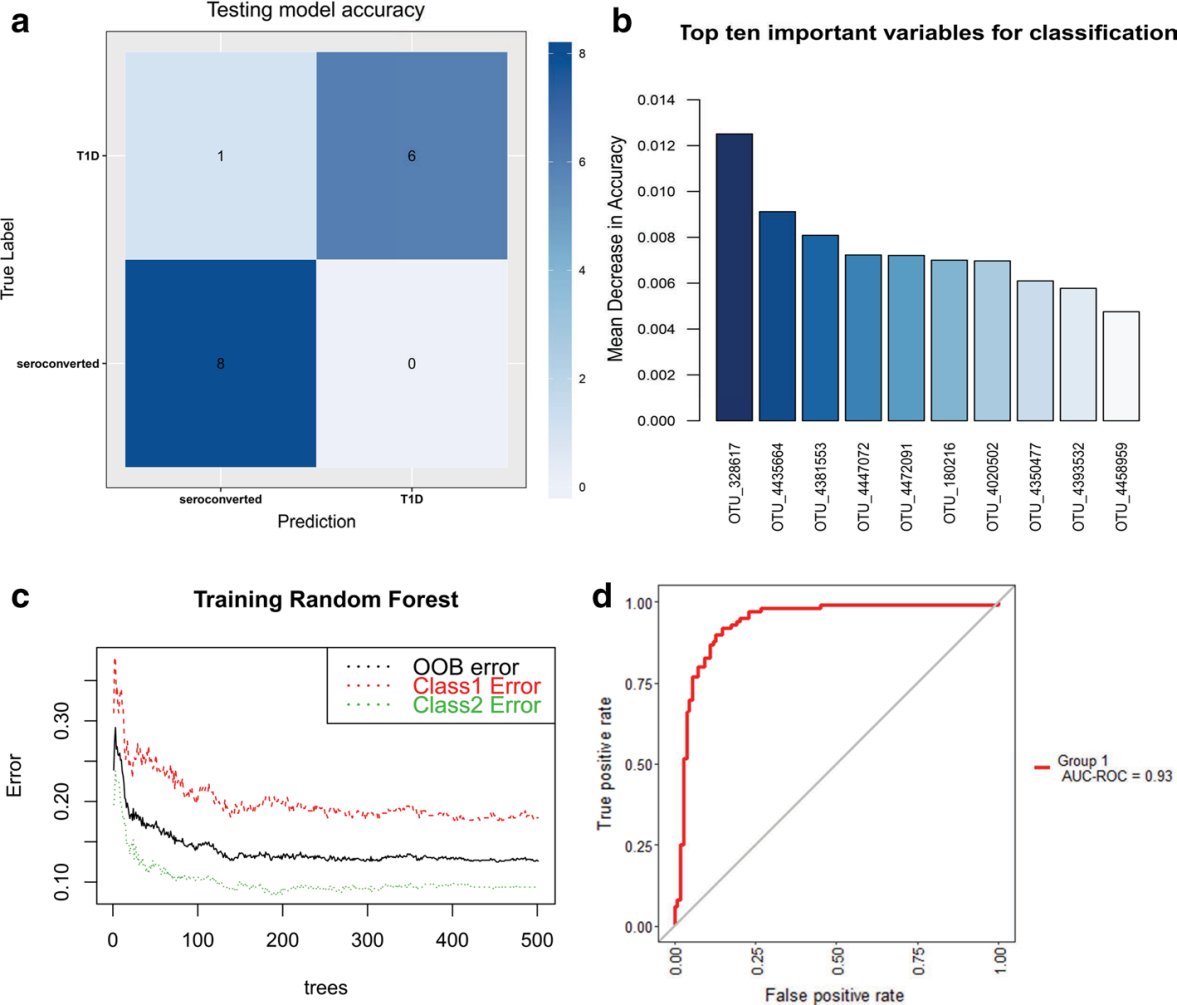
MegaR model prediction results of T1D cohorts using RF. **a** Error rate, **b** confusion matrix, **c** important features, **d** AUC graph

We used MegaR to check if optimizing the threshold and percentage of sample with threshold as well as data split for training and testing improves the model (Fig. 3, Table 2). Our result showed a slight increase in accuracy than obtained from preliminary analysis in all the cases. We validated the improved model using cross validation. The validation accuracy for all the models was within 80- 90% range except for the antibiotic cohort using WGS, for which the cross validation accuracy was 72%.

**Table 2:** Accuracy of optimized RF and cross validation results across three datasets from DIABIMMUNE research group

Dataset	Data type	Optimal model parameter	Model accuracy	95% CI	Cross validation accuracy
Three country cohort	16S	80%, 100T, 20P	0.9028	0.9382–0.8562	0.8685
	WGS	70%, 100T, 10P	0.8864	0.8312–0.9285	**0.8803**
T1D cohort	16S	80%, 5T, 5P	0.9615	0.8686–0.9928	**0.9069**
	WGS	90%, 100T, 10P	0.9481	0.6774–0.9987	0.9036
Antibiotics cohort	16S	70%, 0T, 0P	0.8772	0.8312–0.9285	**0.8643**
	WGS	80%, 10T, 10P	0.7916	0.6502–0.8951	0.7205

Bold numbers represent highest values for the given data set. 16S RNA and WGS data was tested for each of the three data sets. Optimal model parameters are the values used to obtain the highest accuracy for the data set

We also checked if there is any age wise difference between our tool to classify the model. Although the overall performance of the model was within the accuracy of 77% to 90%, the 95% interval was very large (66–95% for 3 years) showing unreliable nature of the model. This could be due to the low number of samples available for building the model.

### Benchmarking

We benchmarked MegaR against MetAML using the T2D [38, 39] and Cirrhosis [40] data sets provided by the MetAML project [28]. Using MegaR, we were able to obtain a slightly higher prediction accuracy for both datasets compared to the results reported by the MetAML project (Table 3). The model parameters used to achieve these results with MegaR were as follows. A threshold of 0.003 was used with a 90% 5 T 5P split. We believe that this slight increase is due to the ability of the MegaR package to fine tune the model parameters to easily optimize the model for each data set.

**Table 3:** Highest observed cross validation accuracies of MegaR and MetAML on T2D and Cirrhosis data sets

Dataset	Program	Accuracy	Percent difference
T2D	MegaR	**0.6683**	0.6509
	MetAML	0.6640	
Cirrhosis	MegaR	**0.8846**	0.8608
	MetAML	0.8770	

Bold values represent the highest obtained accuracy for each dataset

## Conclusions

The MegaR package is an easy to use, versatile tool built with the intent of encouraging the use of machine learning analysis of metagenomic dataset for the purpose of phenotypic prediction and classification. The user-friendly interface allows users to fine tune the model for the specific data set in use in order to maximize the prediction accuracy, therefore increasing the potential functionality of machine learning for these tasks.

For each analysis, MegaR provides various useful metrics in the forms of tables and graphs that allow the user to determine if (1) there is enough available data to build a model, (2) the error rate of the model by more of error graph and confusion matrix, (3) a list of the top 10 most important features identified by the model, which allows researchers to focus on these features for further research or drug development, (4) downloadable figures to be used in further publications.

Our results indicate that the RF model provides the highest accuracy in most metagenomic classification scenarios compared to SVM and GLM. GLM is useful for the examination of 16S rRNA due to the large number of samples compared to WGS data sets, although GLM is less efficient on datasets with high dimensionality. While the standard split criteria in machine learning is 80:10:10 for train:validation:test, we tested various split criteria and, depending on the data, obtained various accuracies. Many machine learning models do not perform well if features are very sparse. As anticipated, removing sparse features expressed in low numbers increased the machine learning model accuracy. Our cross validation of the improved model shows that the models are robust and can be used for prediction with the obtained confidence. In the near future, we plan to test other machine learning classifiers and deep learning methods to increase the prediction accuracy with fast turnaround time.

## Availability and requirements

**Project name**: MegaR.

**Project home page**: https://github.com/BioHPC/MegaR.

**Operating system(s)**: Windows, Mac, and Linux (Platform independent).

**Programming language**: R.

**Other requirements**: R 3.6 or higher. 

**License**: GNU GPL-3. 

**Any restrictions to use by non-academics**: None.

## Data Availability

We mainly used the data sets from DIABIMMUNE microbiome project (https://pubs.broadinstitute.org/diabimmune) [22–24]. The processed dataset for MegaR can be also found under the MegaR project website (https://github.com/BioHPC/MegaR). We also benchmarked MegaR using the T2D [38, 39] and Cirrhosis [40] data sets provided by the MetAML project [28].

## Abbreviations

16S: 16S rRNA sequencing
BIOM: Biological Observation Matrix
CAMDA: Critical Assessment of Massive Data Analysis
GLM: Generalized linear model
HMP: Human microbiome project
IMIDs: Immune-mediated inflammatory diseases
OTU: Operational taxonomic unit
RF: Random forest
SVM: Support vector machine
T1D: Type I diabetes
T2D: Type II diabetes
WGS: Whole genome sequencing
